# Scenario of the spread of the invasive species *Zaprionus indianus* Gupta, 1970 (Diptera, Drosophilidae) in Brazil

**DOI:** 10.1590/S1415-47572010005000080

**Published:** 2010-12-01

**Authors:** Luís Gustavo da Conceição Galego, Claudia Marcia Aparecida Carareto

**Affiliations:** Departamento de Biologia, Universidade Estadual Paulista Júlio de Mesquita Filho, São José do Rio Preto, SPBrazil

**Keywords:** *Zaprionus indianus*, esterase, landscape shape interpolation, Monmonier's maximum difference algorithm

## Abstract

*Zaprionus indianus* was first recorded in Brazil in 1999 and rapidly spread throughout the country. We have obtained data on esterase loci polymorphisms (Est2 and Est3), and analyzed them, using Landscape Shape Interpolation and the Monmonier Maximum Difference Algorithm to discover how regional invasion occurred. Hence, it was apparent that *Z. indianus*, after first arriving in São Paulo state, spread throughout the country, probably together with the transportation of commercial fruits by way of the two main Brazilian freeways, BR 153, to the south and the surrounding countryside, and the BR 116 along the coast and throughout the north-east.

## Introduction

**Figure 1:**
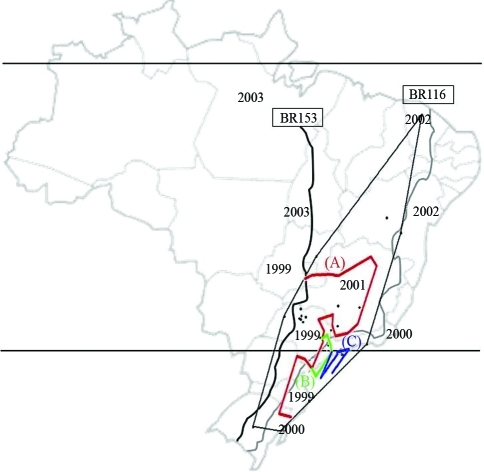
Delaunay triangulation (fine black line) and genetic boundaries in heavy red (A), green (B) and blue (C) lines obtained with the Monmonier maximum difference algorithm. Note that (A) separates the coastal populations, the localities in south-eastern São Paulo state and northern populations from the rest, (B) encompasses the two south-eastern São Paulo populations, thereby isolating them, and (C) isolates coastal populations of the Brazilian south-east and south. The irregular parallel lines across the map of Brazil indicate the BR153 (black line) and BR 116 (gray line) freeways. The numbers indicate the years when *Zaprionus indianus* was first recorded in the various Brazilian states.

*Zaprionus indianus* is an African species, that is now widespread throughout several tropical areas worldwide, probably as a result of the intense commerce of agricultural goods. In Brazil (Figure 1), this drosophilid was first reported by Vilela (1999) in Santa Isabel (São Paulo state), then throughout the state itself (Vilela *et al.*, 2000), and afterwards other neighboring regions (Toni *et al.*, 2001; Tidon *et al.*, 2003). Between 2000 and 2003, the species was progressively observed throughout Brazil as a whole (Castro and Valente, 2001; Santos *et al.*, 2003; Kato *et al.*, 2004; Mata *et al.*, 2004; Loh and Bitner-Mathé, 2005; Mattos-Machado *et al.*, 2005), in Uruguay (Goñi *et al.*, 2001, 2002), and more recently, in Central America and the United States (Linde *et al.*, 2006).

Various tools have been employed for characterizing the species introduced into Brazil, such as alloenzyme polymorphisms (Mattos-Machado *et al.*, 2005; Galego and Carareto, 2007), quantitative traits (David *et al.*, 2006a,b) and chromosome inversions (Ananina *et al.*, 2006). These studies indicated that the founder propagul were numerous. Vilela (1999) proposed that *Z. indianus* was maybe introduced by air transport from Africa. This proposal was thereafter endorsed by Tidon *et al.* (2003). Later, Galego and Carareto (2007) added weight to the concept of African introduction based on data from two polymorphic esterase loci, Est2 and Est3, the first with two alleles (Est2^F^ and Est2^S^), the second with four (Est3^1^, Est3^2^, Est3^3^ and Est3^4^). Furthermore, they proposed that maritime introduction was more probably a result of an increase in the commerce of fruits between Africa and Brazil. Nevertheless, how *Z. indianus* was capable of spreading so rapidly countrywide remains a mystery.

We resorted to a landscape genetics approach as a tool to answer this question. This requires constructing a framework for testing the relative influence of landscape and the environmental features of gene flow and genetic discontinuities (Guillot *et al.*, 2005), as well as that of genetic population structure (Manel *et al.*, 2003; Holderegger and Wagner, 2006). It also provides insights into fundamental biological processes (Storfer *et al.*, 2007), such as metapopulation dynamics, the identification of species distribution across specific geographical and anthropogenic barriers, and population connectivity. Several analyses can be performed using this approach, such as interpolation landscapes (Isaaks and Srivastava, 1989), which permit estimating data at unsampled locations by using a mathematical model of the spatial pattern of sampled values, as well as the Monmonier Maximum Difference algorithm (Monmonier, 1973), for identifying putative genetic barriers across landscapes.

Various molecular markers are applicable in landscape genetics, such as mtDNA (Liepelt *et al.*, 2002), AFLP (Jacquemyn, 2004), microsatellites (Poissant *et al.*, 2005) and allozyme polymorphisms (Hitchings and Beebee, 1997; Michels *et al.*, 2001; Pfenninger, 2002; Arnaud, 2003; Hirao and Kudo, 2004). Since esterases appear to be the most polymorphic loci in Brazilian *Z. indianus* populations (Mattos-Machado *et al.*, 2005; Galego *et al.*, 2006; Galego and Carareto, 2007), Est2 and Est3 loci were chosen for inferring the spreading dynamics of *Z. indianus* regionwise.

## Methods

###  Sampling

Specimens of *Z. indianus* were collected from 2004 to 2007, in 22 localities of Brazil (Table 1), 13 in the state of São Paulo (SP), three in Minas Gerais (MG), two in Rio Grande do Sul (RS), and one each in Santa Catarina (SC), Rio de Janeiro (RJ), Bahia (BA), and Brasilia (DF). Individuals were collected with traps containing enticing baits made up of banana and biological yeast, as described by Galego *et al.* (2006). Figure 1 shows the scatterplot of the locations of the populations sampled, with the enclosing convex polygon overlaid by the map of Brazil. Analysis was restricted to collections with more than 10 individuals. Collected individuals were maintained in mass culture with banana-agar medium. A random sample of 20 flies (10 males and 10 females, all 7 days old) of individuals emerging from eggs ovoposited by females from nature, were used for esterase detection.

###  Polyacrylamide gel electrophoresis and esterase detection

Each individual fly was macerated in 15 μL of Tris-HCl 0.1 M, pH 8.8 (CR Ceron, MSc Dissertation, Universidade de São Paulo, 1988), whereupon the homogenate was applied to a 10% polyacrylamide gel. Electrophoresis was carried out in a Tris-glycine buffer pH 8.8 at 200 V for 3 h. A random sample of 20 individuals (10 males and 10 females) from each population was used. In the case of the EST2 system, which is restricted to males (Galego *et al.*, 2006), only 10 individuals were analyzed. Detection of the esterases (EST) was undertaken as suggested by Galego *et al.* (2006). After detection, the gels were stored as described by Ceron *et al.* (1992).

###  Data analysis

Alloenzyme data were analyzed using the computer software programmes TFPGA version 1.3 (Miller, 1997), Genetic Analyses in Excel (GenAlEx) version 6 (Peakall and Smouse, 2006), and Alleles in Space -AIS- (Miller, 2005). Allele and genotype polymorphic-locus frequencies, observed (H_O_) and expected (H_E_) heterozygosity, and Hardy-Weinberg equilibrium, were all estimated by TFPGA. The estimation of genetic distances (Nei, 1972) and F_ST_ analysis were undertaken with GenAlEx. AIS analysis of Landscape Shape Interpolation (LSI) and the Monmonier Maximum Difference Algorithm (MMDA), was performed to evaluate inter-individual patterns of genetic and geographical variation. The calculated surface for LSI was based on the midpoints of edges derived from Delaunay triangulation (Watson, 1992; Brouns *et al.*, 2003), and the heights on “pseudoslopes” from the genetic and geographical distance matrix (Miller, 2005). The LSI approach visualizes the graphical representation of the pattern of genetic distance across the whole landscape, and is a way of producing a 3-dimensional surface plot where the X and Y axes correspond to geographical locations, whereas surface heights (Z-axes) represent genetic distances. Basically, the figure contains an inferred graphical representation of patterns of diversity across the sampled landscape that (ideally) contains peaks in areas where there are large genetic distances. The initial construction is Delaunay triangulation (Watson, 1992; Brouns *et al.*, 2003) based on connectivity networks of sampling areas and assigning genetic distances, whereupon interpolation procedure (a = 1, grid size = 50 x 50, raw Nei, 1972, genetic distance between points) can be applied.

Furthermore, the building of putative genetic barriers across landscapes, as determined by MMDA, is found in the connectivity network of all the sampled locations used in studies that are generated in three steps by Delaunay triangulation (Watson, 1992; Brouns *et al.*, 2003). The first step is to identify the greatest genetic distance between any 2 locations joined in the connectivity network, thereby forming the initial barrier segment. Secondly, the initial barrier is followed in one direction until encountering either an external edge of the connectivity network or an internal segment previously defined as a barrier segment. In essence, for each extension of the barrier, the movement is in the direction of the greatest genetic distance between locations. Finally, the initial barrier identified in Step 1 is followed in the opposite direction to that taken in Step 2, until, once again, encountering either an external edge of the connectivity network or an internal segment previously defined as a barrier segment.

## Results

The analysis of Est2 allele frequency distribution in Brazilian populations of *Z. indianus* (Table 1) shows fixation of the alleles Est2^S^ in 8 of the 22 populations studied, and Est2^F^ in 3. Est2^S^ frequency was the lowest in Alfenas (0.09), and that of Est2^F^ in Onda Verde and Rio de Janeiro (0.08). The frequency of locus Est3 alleles (Table 1) varied considerably according to geographic location, the least frequent being Est3^3^. Est3^1^ frequency varied from 0 (Ilhabela) to 0.94 (Santa Maria), Est3^4^ from 0.05 (Rio Claro and Porto Alegre) to 0.89 (Ilhabela), and Est3^3^ from 0 (in several localities) to 0.30 (Onda Verde). The frequency of Est3^2^, although not detected in Santa Maria, Onda Verde and Ilhabela, was the highest in Brasília (0.69).

The average observed (H_O_) and expected (H_E_) was greater in Est3 than in Est2 (Table 2). Est3 H_O_ ranged from 0 (Ilhabela) to 0.80 (Onda Verde) and H_E_ from 0.20 (Ilhabela) to 0.70 (Mirassol and Ibirá). The average Est3 H_O_ in populations from São Paulo state (0.54 ± 0.07), and the southern (0.47 ± 0.08) and northern (0.49 ± 0.11) states did not differ significantly (X^2^ = 0.10). On the other hand, Est2 H_O_ ranged from 0 (11 populations) to 0.45 (Brasilia), and H_E_ from 0 (11 populations) and 0.50 (Porto Alegre). The average Est2 H_O_ in populations from São Paulo state (0.08 ± 0.03) and southern regions (0.19 ± 0.06) were not significantly different. Almost all the populations, except those from Ibirá and Maresias, were estimated to be under Hardy-Weinberg equilibrium, with p < 0.05.

Pairwise genetic distance (Nei 1972) and F_ST_ (Weir and Cockerham, 1984) indices differed significantly from zero in several populations (Table S1). About 91% of the pairwise F_ST_ values were significantly different from zero. The overall F_ST_ value was 0.414 (p < 0.001), and the pairwise estimates of F_ST_ ranged from 0.003 (Sud Menucci versus Paraibuna) to 1.000 (Santa Maria versus Poços de Caldas).

**Figure 2:**
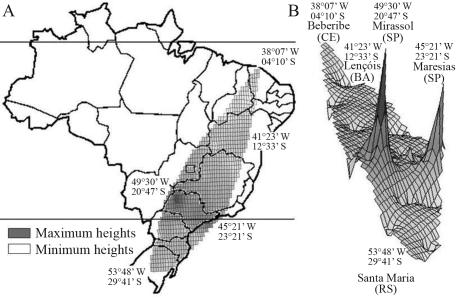
Genetic Landscape Shape Interpolation (GLSI) analysis, using a 50 x 50 grid and a distance weighting parameter of 1. A: polygonal plot of GLSI, overlaid by the map of Brazil. The peaks indicate the localization of the highest genetic variability. B: a 3-dimensional surface-plot view. The dark zones indicate areas with the highest genetic variability. The geographic coordinates of regions with the highest and lowest genetic variability are indicated in the map and 3-dimensional plotting.

Genetic boundaries depicted in Est2 and Est3 data are shown in Figure 1. The first boundary (A) separated the coastal populations (Rio de Janeiro, Maresias, Ilhabela and Florianópolis), the localities in south-eastern São Paulo state (Paraibuna, Itatiba, São Paulo and Rio Claro) and northern populations (Brasília, Jequié, Lençóis and Beberibe), from the rest. The second boundary (B) enclosed Itatiba and São Paulo, thereby isolating both populations. The last (C), isolated Florianópolis, Maresias and Ilhabela and coincided with the geological formation composed of the Serra do Mar Range. Genetic Landscape Shape Interpolation analysis (Figure 2) generated peaks indicating the greatest genetic distances in populations from São Paulo, Itatiba and other localities of south-eastern São Paulo state, decreasing from there in direction to the north and south of Brazil.

## Discussion

Originally from tropical Africa, historical records show that *Z. indianus* arrived in Brazil in 1998 (Vilela, 1999), and quickly spread throughout São Paulo (Vilela *et al.*, 2000), Rio de Janeiro (Loh and Bitner-Mathé, 2005), and the southern (Toni *et al.*, 2001; Castro and Valente 2001) and midwestern (Tidon *et al.*, 2003) states. The remaining Brazilian regions were thereafter rapidly colonized (Santos *et al.*, 2003; Kato *et al.*, 2004; Mata *et al.*, 2004; Mattos-Machado *et al.*, 2005), 5 years after the first records in Para, one of the most northerly states in Brazil (Santos *et al.*, 2003).

The polymorphism displayed by both alloenzyme markers demonstrated a significant geographical genetic structure among the 22 Brazilian populations of *Z. indianus* sampled in this study, as shown by the F_ST_ and Nei (1972) genetic distance values. The Est3 H_O_ values of the Brazilian populations of *Z. indianus* (0.54) were almost the same as the three esterase H_O_ of Indian population loci, each of which harboring 5 alleles, *i.e.*, 0.54 and 0.56 (Parkash *et al.*, 1994) and 0.58 (Parkash and Yadav, 1993), respectively. However, the Est2 H_O_ values from Brazilian populations (0.08) were smaller than an esterase locus with two alleles in Indian populations, viz., 0.17 (Parkash and Yadav, 1993) and 0.33 (Parkash *et al.*, 1994). These differences could be attributed to genetic drift (sampling errors) or the founder effect.

Allele frequencies were employed in the relatively promising, but little used, methodologies of spatial interpolation (Storfer *et al.*, 2007) and the Monmonier algorithm. These approaches could be especially useful in the case of continuously distributed species, by representing allele frequency across a landscape surface, and identifying putative genetic barriers. Normally, mitochondrial DNA markers have been used in these analyses (Dupanloup *et al.*, 2002). By using mtDNA HVRI polymorphism, it was thus possible to infer the action of a past specific barrier hindering gene flow between Italian and Balkanic populations of the European roe deer. Moreover, Manni *et al.* (2004) suggested that the Monmonier algorithm could also be applied in the identification of barriers by using geographical patterns of genetic, morphological and linguistic variation.

The application of these approaches to our data facilitated depicting the graphic pattern of the ratio between genetic and geographic distances (pseudoslope) throughout the sampled regions, with the surface edges corresponding to the highest ratios. All the edges were located in southeastern Brazil, specifically São Paulo state, thereby indicating the higher genetic structuring of these populations, possibly due to both early origin and low gene flow. Historical data reinforce the idea of the earlier arrival of *Z. indianus* in São Paulo state, whereas the 2 highest peaks in the graphical surface, isolated by A and B putative barriers, as inferred by MMDA analysis, suggest population isolation. Based on these clues, analysis of genetic data reinforces the hypothesis that São Paulo state was the center from which *Z. indianus* spread throughout Brazil. On the other hand, the northern and southern populations presented the lowest ratios between genetic and geographic distances, as shown by depressions in the graph-surface. This landscape indicated lower genetic structuring, probably due to a later invasion*.* This scenario agrees with the above-cited historical records.

By identifying 3 boundaries for gene flow through MMDA analysis, a putative scenario of the spread of *Z. indianus* in Brazil can be visualized (Figure 1). Boundary A separates the coastal populations from the remainder, boundary B isolates the towns of São Paulo and Itatiba, both located very close to Valinhos, where *Z. indianus* was first observed, whereas boundary C corresponds to a natural geological barrier, the Serra do Mar, a 1500 km long mountain range extending from Espírito Santo to Santa Catarina states. These boundaries separate two of the main highways in Brazil, the BR153 and BR116. The first is an important route for commercial interchange with inland Brazil (Confederação Nacional de Transportes a), whereas the second is coastal (Confederação Nacional de Transportes b). A similar manner of diffusion, due to the fruit trade, may have occurred in the Palearctic region (Yassin *et al.*, 2009). However, in the Americas the spread was extremely fast (about six years, from São Paulo to Florida), in contrast to the Palearctic region, where it took more than 40 years for *Z. indianus* to spread from India to Egypt. The great difference in the pace of spread between Brazil/USA and India/Egypt can be attributed to the more developed freeway networks in Brazil than in the Palearctic region.

These findings suggest that the spreading of *Z. indianus* occurred from São Paulo, the state where commercial highway traffic is the heaviest, to the north and south of Brazil by way of both the BR153 and the BR116 highways. The landscape genetics approach hereby applied for characterizing the genetic structure of populations from an initial colonizer species soon after its introduction, as well as its relevance in offering the possibility of determining the source of invasion, and demographic parameters of the species, also offers a unique opportunity for accompanying the evolutionary dynamics of the invader species over time.

## Supplementary Material

The following online material is available for this article:

Table S1 Pairwise values of F_ST_ and genetic distance between the Brazilian populations of *Zaprionus indianus*.

This material is available as part of the online article from http://www.scielo.br/gmb.

## Figures and Tables

**Table 1:** Geographical coordinates of the *Zaprionus indianus* populations sampled and allele frequencies of Est3 and Est2 esterase loci. 1: Est3^1^; 2: Est3^2^; 3: Est3^3^; 4: Est3^4^; S: Est2^S^; F: Est2^F^; H_O_: observed heterozygosity; H_E_: expected heterozygosity; ne: not evaluated. *Mattos-Machado *et al. (*2005).

		Est3							Est2			
Locality	Latitude	1	2	3	4	H_O_	H_E_		S	F	H_O_	H_E_
State of São Paulo (SP)												
Mirassol	49°30' W/20°47' S	0.40	0.22	0.10	0.28	0.60	0.70		0.60	0.40	0.30	0.35
Onda Verde	49°30' W/20°62' S	0.30	0.00	0.30	0.40	0.80	0.66		0.92	0.08	0.17	0.15
São José do Rio Preto	49°22' W/20°49' S	0.34	0.12	0.08	0.46	0.12	0.12		0.00	1.00	0.00	0.00
Itatiba	46°50' W/23°00' S	0.16	0.21	0.16	0.47	0.79	0.68		0.36	0.64	0.18	0.46
Ilhabela	45°21' W/23°46' S	0.00	0.00	0.11	0.89	0.00	0.20		1.00	0.00	0.00	0.00
Paulo de Faria	49°30' W/20°62' S	0.46	0.12	0.13	0.29	0.50	0.67		0.31	0.69	0.22	0.49
São Paulo	46°50' W/23°31' S	0.18	0.23	0.09	0.50	0.64	0.66		0.42	0.58	0.17	0.49
Paraibuna	45°41' W/23°26' S	0.44	0.21	0.21	0.14	0.62	0.67		1.00	0.00	0.00	0.00
Maresias	45°21' W/23°21' S	0.50	0.17	0.04	0.29	0.42	0.63		1.00	0.00	0.00	0.00
Rio Claro	44°08' W/22°43' S	0.65	0.20	0.10	0.05	0.70	0.52		1.00	0.00	0.00	0.00
Ibirá	49°14' W/21°04' S	0.42	0.29	0.12	0.17	0.42	0.70		1.00	0.00	0.00	0.00
Olímpia	48°54' W/20°44' S	0.35	0.35	0.00	0.30	0.67	0.66		0.00	1.00	0.00	0.00
Sud Menucci	50°55' W/20°41' S	0.45	0.25	0.15	0.15	0.70	0.69		1.00	0.00	0.00	0.00
Southern States												
Porto Alegre (RS)	51°13' W/30°01' S	0.33	0.62	0.00	0.05	0.45	0.51		0.53	0.47	0.35	0.50
Santa Maria (RS)	53°48' W/29°41' S	0.94	0.00	0.00	0.06	0.12	0.12		1.00	0.00	0.00	0.00
Florianópolis (SC)	48°32' W/27°35' S	0.14	0.28	0.05	0.53	0.60	0.62		0.82	0.17	0.36	0.30
Alfenas (MG)	46°10' W/21°20' S	0.32	0.03	0.03	0.62	0.29	0.51		0.09	0.91	0.18	0.16
Belo Horizonte (MG)	43°56' W/19°55' S	0.52	0.16	0.00	0.32	0.56	0.60		0.71	0.29	0.29	0.41
Córrego Danta (MG)	45°55' W/19°24' S	0.18	0.28	0.00	0.54	0.53	0.60		0.00	1.00	0.00	0.00
Poços de Caldas (MG)*	46°33' W/21°47' S	0.00	0.50	0.50	0.00	ne	ne		ne	ne	ne	ne
Rio de Janeiro (RJ)	43°12' W/22°54' S	0.50	0.17	0.23	0.10	0.73	0.66		0.92	0.08	0.12	0.12
Northern States												
Brasília (DF)	47°55' W/15°46' S	0.11	0.69	0.00	0.20	0.38	0.47		0.32	0.68	0.45	0.35
Jequié (BA)	40°04' W/13°51' S	0.30	0.45	0.10	0.15	0.60	0.68		1.00	0.00	0.00	0.00
Lençóis (BA)*	41°23' W/12°33' S	0.05	0.57	0.38	0.00	ne	ne		ne	ne	ne	ne
Beberibe (CE)*	38°07' W/04°10' S	0.21	0.45	0.24	0.10	ne	ne		ne	ne	ne	ne

**Table 2:** Means ( $\bar{X}$) and standard-errors (SE) of observed (H_O_) and expected (H_E_) heterozygosity in Brazilian populations of *Zaprionus indianus* and chi-squared comparison (*x*^2^). ne: not evaluated.

Geographic region		Est2 ( $\bar{X}$ ± *SE*)	Est3 ( $\bar{X}$ ± *SE*)	*x*^2^
São Paulo state (SP)	H_O_	0.08 ± 0.03	0.54 ± 0.07	5.05*
	H_E_	0.15 ± 0.06	0.58 ± 0.05	4.18*
	*x*^2^	0.42	0.04	
Southern (S)	H_O_	0.19 ± 0.06	0.47 ± 0.08	1.19
	H_E_	0.21 ± 0.07	0.52 ± 0.07	1.25
	*x*^2^	0.02	0.03	
Northeast (N)	H_O_	ne	0.49 ± 0.11	ne
	H_E_	ne	0.57 ± 0.10	ne
	*x*^2^	ne	0.02	
Total	H_O_	0.13 ± 0.03	0.51 ± 0.04	6.35**
	H_E_	0.17 ± 0.04	0.56 ± 0.04	5.93*
	*x*^2^	0.25	0.10	
*SPXS*	*x*^2^	6.37**	1.04	
*SPXN*	*x*^2^	Ne	0.51	
*SXN*	*x*^2^	Ne	ne	

*p <0.05; **p <0.01; ***p <0.001.
