# Supplementary material for: Scenario of the spread of the invasive species Zaprionus indianus Gupta, 1970 (Diptera, Drosophilidae) in Brazil
Source: Genet Mol Biol. 2010 Dec 1;33(4):767–73. doi: 10.1590/S1415-47572010005000080 (PMC3036140; doi:10.1590/S1415-47572010005000080)
Supplement: Table S1 — Pairwise values of FST and genetic distance between the Brazilian populations of Zaprionus indianus. [file gmb-33-4-767-suppl1.pdf]

Table S1 - Pairwise values of F_{ST} (Weir and Cockerhan 1984), above diagonal, and genetic distance (Nei 1972), below diagonal, between the Brazilian populations of *Zaprionus indianus.* Significant values are indicated in **bold** typeface. *São Paulo state:* IB (Ibirá), IL (Ilhabela), IT (Itatiba), MA (Maresias), MI (Mirassol), OL (Olímpia), OV (Onda Verde), PF (Paulo de Faria), PR (Paraibuna); RC (Rio Claro); SJ (São José do Rio Preto), SU (Sud Menucci) and SP (São Paulo); *other states of Southeast:* AL (Alfenas), BH (Belo Horizonte), CD (Córrego Danta), PC* (Poços de Caldas) and RJ (Rio de Janeiro); *South states:* FL (Florianópolis), PA (Porto Alegre) and SM (Santa Maria); and *North and Mideast states:* BR (Brasília), JE (Jequié), LE* (Lençóis) and BE* (Beberibe). *Machado *et al.* 2005.

	IB	IL	IT	MA	MI	OL	OV	PF	PR	RC	SJ	SU	SP	AL	BH	CD	RJ	BR	FL	PA	SM	JE	LE*	BE*	PC*
**IB**	*****	0.226	0.460	**0.015**	0.099	0.767	0.086	0.458	**0.013**	**0.039**	0.797	**0.006**	0.425	0.727	0.105	0.798	**0.033**	0.511	0.100	0.232	0.117	**0.013**	0.704	0.581	0.752
**IL**	0.296	*****	0.354	0.195	0.267	0.827	0.111	0.527	0.281	0.376	0.734	0.274	0.388	0.559	0.266	0.716	0.319	0.664	0.070	0.560	0.439	0.263	0.963	0.851	0.948
**IT**	0.272	0.299	*****	0.452	0.181	0.160	0.336	0.075	0.498	0.562	0.116	0.493	**0.011**	0.085	0.260	0.107	0.449	0.182	0.256	0.282	0.619	0.473	0.713	0.605	0.718
**MA**	**0.015**	0.285	0.277	*****	0.093	0.753	0.076	0.428	**0.031**	**0.038**	0.762	**0.019**	0.411	0.667	0.067	0.780	**0.045**	0.570	0.101	0.287	0.078	0.053	0.830	0.654	0.879
**MI**	0.108	0.233	0.068	0.110	*****	0.382	0.117	0.148	0.115	0.128	0.394	0.105	0.142	0.352	**0.023**	0.411	0.087	0.287	0.111	0.130	0.176	0.126	0.703	0.530	0.749
**OL**	0.504	0.634	0.133	0.507	0.245	*****	0.741	0.089	0.795	0.770	**0.028**	0.777	0.120	0.089	0.444	**0.026**	0.706	0.162	0.630	0.318	0.767	0.772	0.734	0.597	0.801
**OV**	0.060	0.118	0.179	0.059	0.068	0.449	*****	0.385	0.080	0.147	0.677	0.090	0.345	0.574	0.128	0.720	0.083	0.608	0.099	0.399	0.206	0.133	0.830	0.710	0.787
**PF**	0.278	0.386	**0.024**	0.273	0.064	0.099	0.203	*****	0.466	0.456	0.075	0.457	**0.040**	0.081	0.183	0.115	0.385	0.227	0.378	0.249	0.449	0.500	0.788	0.600	0.813
**PR**	**0.012**	0.344	0.286	**0.031**	0.115	0.519	0.058	0.282	*****	**0.023**	0.817	**0.003**	0.464	0.765	0.122	0.846	**0.009**	0.569	0.160	0.267	0.094	**0.035**	0.704	0.590	0.722
**RC**	**0.046**	0.483	0.334	**0.048**	0.133	0.538	0.099	0.301	**0.030**	*****	0.813	**0.017**	0.500	0.770	0.102	0.856	**0.021**	0.605	0.233	0.272	**0.030**	0.075	0.779	0.612	0.827
**SJ**	0.517	0.580	0.116	0.512	0.249	**0.027**	0.422	0.093	0.529	0.559	*****	0.807	0.099	**0.029**	0.446	**0.022**	0.725	0.255	0.615	0.433	0.792	0.826	0.843	0.696	0.862
**SU**	**0.006**	0.343	0.286	**0.020**	0.112	0.513	0.063	0.280	**0.003**	**0.023**	0.526	*****	0.452	0.749	0.105	0.828	**0.014**	0.551	0.146	0.249	0.083	**0.028**	0.718	0.588	0.754
**SP**	0.258	0.315	**0.003**	0.260	0.056	0.118	0.180	**0.013**	0.273	0.311	0.110	0.270	*****	0.077	0.205	0.093	0.410	0.154	0.256	0.223	0.542	0.441	0.719	0.577	0.752
**AL**	0.464	0.462	0.067	0.447	0.199	0.070	0.351	0.060	0.483	0.521	**0.038**	0.478	0.064	*****	0.378	**0.047**	0.688	0.315	0.498	0.477	0.738	0.773	0.952	0.769	0.969
**BH**	0.103	0.240	0.107	0.090	**0.010**	0.283	0.069	0.086	0.111	0.115	0.284	0.104	0.088	0.226	*****	0.482	0.094	0.408	0.140	0.210	0.104	0.159	0.853	0.621	0.912
**CD**	0.525	0.579	0.115	0.528	0.260	**0.027**	0.447	0.112	0.548	0.587	**0.022**	0.543	0.110	**0.050**	0.305	*****	0.772	0.172	0.572	0.382	0.867	0.800	0.768	0.650	0.819
**RJ**	**0.038**	0.212	0.217	**0.045**	0.058	0.436	**0.034**	0.203	**0.027**	**0.038**	0.444	**0.030**	0.203	0.402	0.056	0.469	*****	0.539	0.188	0.247	0.076	0.064	0.711	0.579	0.722
**BR**	0.320	0.489	0.072	0.354	0.124	0.134	0.302	0.085	0.345	0.392	0.172	0.340	0.062	0.166	0.185	0.142	0.281	*****	0.391	0.095	0.723	0.446	0.423	0.397	0.532
**FL**	0.077	0.106	0.137	0.082	0.056	0.394	**0.044**	0.188	0.101	0.148	0.386	0.097	0.136	0.308	0.067	0.371	0.077	0.214	*****	0.265	0.328	0.104	0.739	0.628	0.786
**PA**	0.173	0.382	0.102	0.203	**0.048**	0.221	0.182	0.097	0.189	0.213	0.266	0.183	0.083	0.249	0.086	0.253	0.134	0.052	0.130	*****	0.377	0.188	0.439	0.360	0.559
**SM**	0.211	0.748	0.434	0.170	0.209	0.624	0.182	0.368	0.185	0.097	0.637	0.171	0.402	0.610	0.167	0.687	0.109	0.533	0.254	0.343	*****	0.186	0.951	0.717	1.000
**JE**	**0.013**	0.333	0.279	0.052	0.120	0.510	0.079	0.296	**0.032**	0.086	0.534	**0.027**	0.266	0.488	0.127	0.530	0.052	0.293	0.080	0.156	0.295	*****	0.601	0.523	0.662
**LE**	0.557	0.735	0.448	0.601	0.439	0.572	0.541	0.467	0.560	0.617	0.597	0.566	0.445	0.601	0.499	0.588	0.516	0.418	0.514	0.403	0.757	0.533	*****	0.077	**0.035**
**BE**	0.514	0.672	0.408	0.540	0.390	0.523	0.494	0.412	0.518	0.554	0.543	0.519	0.400	0.535	0.432	0.541	0.470	0.406	0.470	0.377	0.667	0.504	0.516	*****	0.148
**PC**	0.571	0.736	0.451	0.615	0.450	0.590	0.534	0.474	0.566	0.631	0.603	0.577	0.454	0.608	0.513	0.603	0.521	0.452	0.527	0.437	0.771	0.552	0.508	0.528	*****
